# Cyanobacterial Cell Lineage Analysis of the Spatiotemporal *hetR* Expression Profile during Heterocyst Pattern Formation in *Anabaena* sp. PCC 7120

**DOI:** 10.1371/journal.pone.0007371

**Published:** 2009-10-12

**Authors:** Hironori Asai, Shunsuke Iwamori, Kentaro Kawai, Shigeki Ehira, Jun-ichi Ishihara, Kazuyuki Aihara, Shuichi Shoji, Hideo Iwasaki

**Affiliations:** 1 Department of Electrical Engineering and Biological Science, Waseda University (TWIns), Tokyo, Japan; 2 PRESTO, Japan Science and Technology Agency (JST), Tokyo, Japan; 3 Department of Electrocic and Photonic Systems, Waseda University, Tokyo, Japan; 4 Department of Biological Science, Chuo University, Kasuga, Tokyo; 5 Institute of Industrial Science, The University of Tokyo, Tokyo, Japan; 6 ERATO Aihara Complexity Modelling Project, JST, Tokyo, Japan; Texas A& M University, United States of America

## Abstract

Diazotrophic heterocyst formation in the filamentous cyanobacterium, *Anabaena* sp. PCC 7120, is one of the simplest pattern formations known to occur in cell differentiation. Most previous studies on heterocyst patterning were based on statistical analysis using cells collected or observed at different times from a liquid culture, which would mask stochastic fluctuations affecting the process of pattern formation dynamics in a single bacterial filament. In order to analyze the spatiotemporal dynamics of heterocyst formation at the single filament level, here we developed a culture system to monitor simultaneously bacterial development, gene expression, and phycobilisome fluorescence. We also developed micro-liquid chamber arrays to analyze multiple *Anabaena* filaments at the same time. Cell lineage analyses demonstrated that the initial distributions of *hetR::gfp* and phycobilisome fluorescence signals at nitrogen step-down were not correlated with the resulting distribution of developed heterocysts. Time-lapse observations also revealed a dynamic *hetR* expression profile at the single-filament level, including transient upregulation accompanying cell division, which did not always lead to heterocyst development. In addition, some cells differentiated into heterocysts without cell division after nitrogen step-down, suggesting that cell division in the mother cells is not an essential requirement for heterocyst differentiation.

## Introduction

The multicellular (filamentous) cyanobacterium, *Anabaena* sp. PCC 7120, differentiates into cells named heterocysts, which are specialized for nitrogen fixation every ∼10 cells along the filament under nitrogen-deprived conditions [reviewed by 1]–[4]. Genetic dissection has identified several genes important for heterocyst patterning. Expression of the *hetR* gene encoding a protein harboring both DNA-binding [5] and serine protease activities [6] is essential for heterocyst development [7], [8]. The *hetR* gene is induced by nitrogen deprivation [7] and becomes localized to heterocysts [9]. On the other hand, the *patS* gene encodes a small peptide to inhibit heterocyst formation and is also induced by nitrogen fixation and localized to heterocysts [10], [11]. Interestingly, it has been proposed that the HetR protein activates its own transcription and *patS* gene expression, while the PatS peptide inhibits HetR's function by direct association [2], [5], [9]. The *hetN* gene product is also a known inhibitor of *hetR* expression [12], [13]. Although HetN is not essential for *de novo* heterocyst pattern formation, it appears to be important for the maintenance of the pattern at a later stage [12]. This type of combination of the negative and positive feedback loops with a possible diffusible inhibitor is reminiscent of Turing instability dynamics for regular pattern generation [14], although this and other possibilities have not yet been well validated experimentally.

Most studies on heterocyst patterning were based on statistical analysis using cells collected at different times from a liquid culture. However, for better understanding of the spatiotemporal dynamics underlying the heterocyst pattern formation, detailed quantitative time-lapse observation of heterocyst development in whole identical bacterial filaments grown under the microscope is necessary. For example, the position of *de novo* proheterocysts is considered to be stochastically selected and is thereafter established through interactions between cells, including lateral inhibition [1]. However, it is not known whether stochastic (and/or cell-cycle-dependent) fluctuations in some intracellular activities, such as basal *hetR* expression, at the nitrogen step-down have some impact on the selection of the proheterocyst positions. Considering the autoregulatory property of *hetR* expression [9], an initial fluctuation in *hetR* expression could be enhanced through a subsequent positive feedback process and may affect the selection of proheterocyst positions by lateral inhibition. In this case, the proheterocyst positions would somehow be predetermined or dependent on stochastic fluctuations in the initial conditions. To address these questions, we developed a combined monitoring and culture system that enabled us to observe morphological changes, *hetR* expression profile, and phycobilisome fluorescence from individual filaments during the course of heterocyst development. Moreover, we developed a microelectromechanical system (MEMS)-aided micro-liquid chamber array to analyze simultaneously developmental dynamics from multiple *Anabaena* filaments.

Our cell lineage analyses demonstrated that the initial distributions of *hetR::gfp* and phycobilisome fluorescence signals at nitrogen step-down were not correlated with the resulting distribution of developed heterocysts. We also observed a transient activation of *hetR* expression that did not lead to differentiation. These observations are more consistent with a stochastic rather than a predetermined selection of leading (primary) heterocyst positions *via* dynamic interactions between cells. We also observed cells that differentiated into heterocysts without cell division after nitrogen step-down, suggesting that cell division among mother cells is not an essential requirement for heterocyst differentiation.

## Results and Discussion

### Simultaneous microscopic monitoring of *hetR* gene expression, phycobilisome fluorescence, morphology, and cell lineage analysis

**Figure 1:**
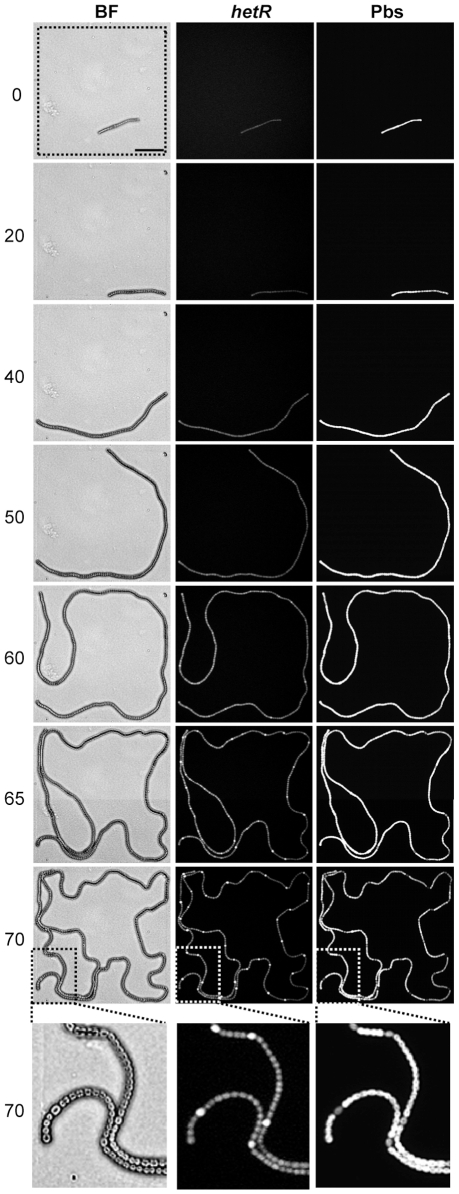
Heterocyst differentiation in microelectromechanical system-assisted liquid microchambers. Spatiotemporal dynamics in morphological changes (BF, bright field), *hetR* promoter (P*hetR*) activity monitored with a transcriptional *gfp* fusion reporter (*hetR*), and phycobilisome fluorescence (Pbs) in an individual *Anabaena* filament at the indicated times after nitrogen step-down. The dashed square at the top indicates the space of a microchamber. The bottom panel (taken at 70 h) shows a magnification of part of the filament.

To investigate quantitative spatiotemporal dynamics during the course of heterocyst development, we analyzed simultaneously morphological changes, *hetR* gene expression profile, and phycobilisome fluorescence patterns in a single *Anabaena* filament under the microscope. For such prolonged observation and culture, we needed avoidance of any three-dimensional bacterial growth that would occur using microchamber arrays. Therefore, we developed a microchamber array made of combined nitrogen-deficient (BG-11_0_) agar containing multiple microwells (200×200×8 µm each) with a silicon mold fabricated by MEMS technology (Figure S1A, B). *Anabaena* cells harboring a P*hetR::gfp* reporter gene were grown in BG-11 liquid medium containing nitrate with aeration with air (normal CO_2_), followed by four washes with BG-11_0_ media lacking combined nitrogen. A small aliquot (∼10 µl) of diluted cell suspension was placed on the bottom of a 35 mm dish, and then covered with the microchamber well so that cells started cell differentiation within a microenvironment (Figure 1, Supporting Movies S1 and S2). Under this condition, cells were able to move and grow within the 200×200 µm space without cell distortion, and the distance to the objective lens was kept constant to avoid changing focus. Furthermore, using automated programming, we were able to monitor simultaneously cell differentiation processes from at least six individual *Anabaena* filaments.

**Figure 2:**
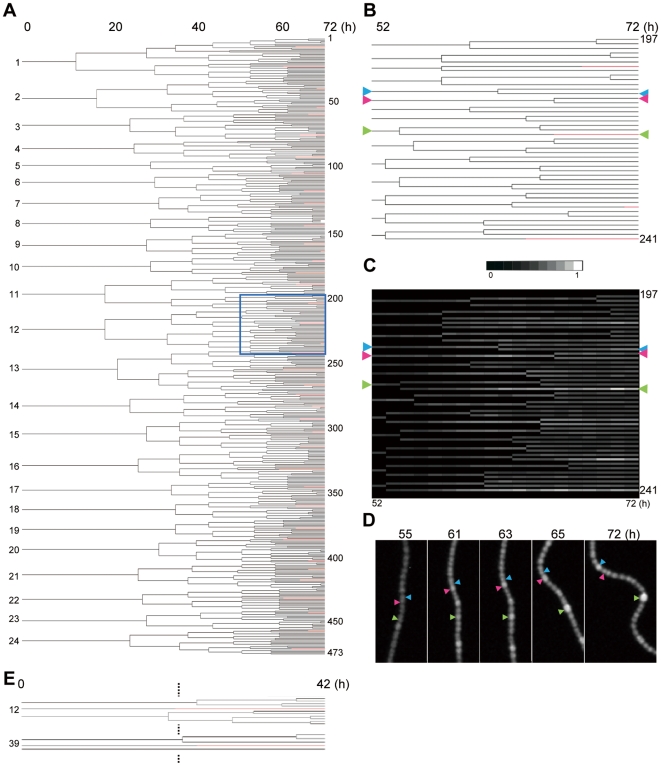
Cell lineage of an individual *Anabaena* filament. (A) The cell lineage analysis representing spatiotemporal profiles of cell division (branches) and heterocyst differentiation (red) in the bacterial filament shown in Figure 1. For a magnified view, see Figure S2A. The horizontal axis shows time (h) after nitrogen step-down. (B, C) Magnification of a part of the *Anabaena* cell lineage shown in the blue square in panel A (B) and the same lineage superimposed with the spatiotemporal dynamics of P*hetR::gfp* signals (C). For a full scale image, see Figure S3A. Arrowheads indicate some cells showing different *hetR* expression dynamics (see text). (D) Micrographs of P*hetR::gfp* expression patterns from the corresponding part of the bacterial filament. (E) Cells that differentiated into heterocysts without cell divisions (numbers 12 and 39). These are parts of the complete cell lineage shown in Figure S2D.

The observed information was integrated into “*Anabaena* cell lineages” representing timing and positions of cell division and heterocyst differentiation (Figures 2A–C; S2A, B; S3A, B). Because an increase in the P*hetR::gfp* signal was not always followed by heterocyst induction (see below), the timing of cell differentiation was determined by any of the following factors: (i) reduction in phycobilisome fluorescence, (ii) brighter P*hetR::gfp* signals or (iii) beginning of cell expansion. Under the microchamber conditions, heterocyst differentiation became visible at 60 h after nitrogen reduction, which was much slower than that in liquid media (Figure S4, under 1% CO_2_) and most previous reports [e.g., 15]. This was possibly caused by poor gas exchange in the solid agar plate lacking aeration and/or the relatively lower CO_2_ concentration. Alternatively, repetitive irradiation of excitation light onto multi-point chambers might be harmful to cell growth. When cells were placed beneath the solid media on the bottom of the plastic dish without microchambers, we were hardly able to observe complete cell lineages in individual filaments, as cells elongated out of the microscopic field. More seriously, the cells were often distorted to be fragmented and even bleached after abnormal expansion of cells, possibly due to some stresses with limited free-moving space. Nevertheless, we could observe only a few filaments with lesser irradiation of excitation lights and without multi-point analysis (single-filament observation per experiment) during the pilot experiments. They developed heterocyst more rapidly after nitrogen step-down, as shown in Movies S3 and S4 (for cell lineage analysis, Figures 2E; S2C, D). The filament shown in Movie S3 was grown without observation of phycobilisome fluorescence and differentiated heterocysts from hour 29 after nitrogen step-down (for cell lineage analysis, see Figure S2C and S3B), while cells shown in Movie S4 differentiated from hour 22 without fluorescence observations. However, these cells were exceptional and much more filaments show abnormal growth as described above. In contrast, when cells were grown under microchamber conditions with observation of both GFP and phycobilisome fluorescence signals for 6 distinct filaments at the same time (Movies S1 and S2), 6-times more irradiation of excitation light for the miscroscopic stage was required compared with a single filament analysis. Note that despite differences in growth conditions (liquid with aeration in the presence of 1% CO_2_ with lesser repetitive irradiation of strong excitation light for Figure S4; microchambers with much irradiations under normal CO_2_ condition for Figure S2A and S2B; solid agar with lesser irradiation under normal CO_2_ condition for Movies S2C and S2D) and growth rate (Figure S5C), the resulting heterocyst patterns were essentially the same to each other as those reported previously (Figure 1, Figure S4, Movies S1 to [Supplementary-material pone.0007371.s009] [Supplementary-material pone.0007371.s010] S4).

### Division of mother cells is not essential for heterocyst formation

The administration of cell division inhibitors and the overproduction of the cell-division-related proteins, SulA and MinC, have been shown to suppress heterocyst development [16], [17]. Thus, heterocyst formation is considered to be tightly coupled to the control of the cell-division cycle. Consistent with this, initial heterocyst differentiation started around 30 h and 10 h after starting logarithmic growth in microchambers (Figure S5A, C) or solid agar (Figure S5B, C) conditions, respectively. Nevertheless, in an *Anabaena* filament grown beneath solid media without microchambers, we found that two vegetative cells differentiated into heterocysts without division during synchronous development (Movie S4, cells numbered 12 and 39 at hour 0 after nitrogen step-down shown in Figures 2E and S2D). Thus, cell division of mother cells is not an essential requirement to differentiate into heterocyst after nitrogen step-down. Note that this does not mean that cell division is not required for heterocyst differentiation. Instead, cell division might be important in causing fluctuations in some intracellular activities in dividing and even nondividing cells, which would affect the differentiation processes (see below).

### Presumable determination of leading heterocysts through dynamic cell–cell interactions before commitment

It is not known whether initial fluctuations in some intracellular activities at the nitrogen step-down affect the selection of proheterocyst positions. Therefore, we examined whether the position of a ‘leading’ heterocyst, defined as a heterocyst appearing first in the cell lineage or during early differentiation, was dependent on the initial conditions at nitrogen step-down under microchamber conditions. We examined two physiological parameters, P*hetR::gfp* signals and phycobilisome fluorescence. Upregulation of *hetR* is essential for heterocyst differentiation, and phycobilisome fluorescence is downregulated during heterocyst maturation. Considering the autoregulation of *hetR* gene expression [9], initial fluctuations in basal *hetR* expression could be enhanced through a subsequent positive feedback process and may affect the selection of proheterocyst positions by lateral inhibition. Cell lineage analysis based on time-lapse observation enabled us to validate this possibility. Because we observed cells every hour under the microscope, the numbers of ‘leading (first) heterocysts’ ranged from one to four under our experimental conditions (Figure S2A, B).

**Figure 3:**
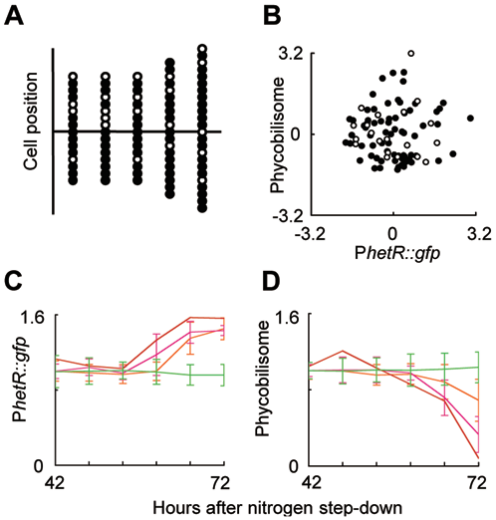
Initial condition-independent selection of leading heterocyst positions and dynamic *hetR* expression profiles. (A) The distributions of cells at the beginning of nitrogen step-down, which generated progenies differentiating into leading heterocysts, are indicated by open circles. The other cells are shown by filled circles. (B) Scatter plot of P*hetR::gfp* and phycobilisome fluorescence signals for cells whose daughter cells differentiated into leading or very early heterocysts (open circle) and the other cells (filled circle) at the beginning of nitrogen step-down. No statistically significant difference was found between the two cell groups for both fluorescence signals using two-sided Student's *t*-test (five filaments with 92 cells). (C, D) Transition P*hetR::gfp* (C) and phycobilisome (D) fluorescence signals in an individual *Anabaena* filament during the course of heterocyst formation from 42–72 h after nitrogen step-down. Cells were categorized into four groups whose progenies (or themselves) differentiated into heterocyst(s): (1) at 62 h after nitrogen step-down (the leading heterocyst, red); (2) at 63–65 h (during the transition state, magenta); (3) at 66–72 h (after establishment of regular patterns, orange) and (4) the remaining vegetative cells at 72 h (green). For data shown in panel C and D, fluorescence intensity was normalized globally so that the mean value per cell was 1.0.

We analyzed five relatively short filaments, starting from 16–24 cells at the time of nitrogen step-down. Although a tendency for heterocyst formation at the termini was observed for these five, the positions of the initial heterocysts were not always distributed regularly (Figure 3A). Terminal heterocysts are already known to appear frequently in short filaments [18]. Moreover, the results represented in Figure 3B showed that, at hour 0 after nitrogen step-down, there was no statistical significance in the magnitude of phycobilisome and P*hetR::gfp* fluorescence signals between cells that were progenitors of the leading heterocysts and cells that were not (Student's *t*-test, *P*>0.1 for both fluorescence signals). This result makes it unlikely that the leading heterocyst positions were already determined at the time of nitrogen step-down).

To confirm this proposition in more detail at the single-filament level, we examined the dynamic profiles of P*hetR::gfp* and phycobilisome fluorescence signals in an individual *Anabaena* filament (Figures 3C, 3D, and S6) from the progenitor cells that generated the leading heterocysts (red), the succeeding heterocysts that differentiated before (magenta) or after (orange) typical regular heterocyst patterning was established, or cells that still remained vegetative at 74 h after nitrogen step-down (green). A statistically significant difference in P*hetR::gfp* signals between those cells becoming heterocysts and other cells emerged around 60 h after nitrogen step-down for the first time (Figure S6), whereas downregulation of phycobilisome fluorescence started from 66 h (Figure S6). As shown in Figures 2C and 2D, the cell marked with a green arrowhead exhibited enhanced P*hetR* activity from 56 h after nitrogen deprivation, and differentiated into a heterocyst. However, such an increase in the level of P*hetR* activity did not always give rise to heterocyst differentiation. For example, the cell marked with a red arrowhead in Figures 2C and 2D showed a transient increase in *hetR* promoter activity from 57–63 h after nitrogen step-down, followed by a decrease in the P*hetR::gfp* signal accompanied by cell division around hour 67. We found at least thirteen cells that showed such transient *hetR* upregulation without heterocyst formation in the same cell filament shown in Figure S2A. Because transient *hetR* induction and downregulation were often accompanied by cell division in most of them, *hetR* expression and differentiation are likely to be suppressed at a certain phase of the cell cycle. Although cell division is asynchronous in our experimental conditions (Figure S2), two sibling cells would be relatively more synchronous in their cell cycles. Interestingly, there was a tendency that transiently *hetR-*expressing cell took somehow longer time to divide by ∼1 h at average than its sibling cell from division of their mother cell (Figure S7A). Therefore, even without leading to heterocyst differentiation, such a transient increase in *hetR* transcription may slightly delay the cell division, as is much more evident for heterocyst forming cells that do not divide anymore.

Next, we extracted total 29 sets of sibling cells from which one termed h-cell differentiated to heterocyst without further cell division, while the other termed v-cell remained to be vegetative to undergo cell division from the lineage shown in Figure S2A (24 sets termed group A, microchambers) and S2C (5 sets termed group B, solid media). As shown in Figure S7B and C, after division of each mother cell, the v-cell underwent cell division at 9.8±1.9 h (group A) or 12.0±0.7 h (group B), while the h-cell differentiated into heterocyst at 10.8±2.4 h (group A) or 12.5±2.3 h (group B). Figure S7D shows normalized data so that time 0 and 1 are defined as the timing of cell division in each mother cell and each v-cell, respectively. These results indicate that the timing of cell differentiation peaked around the cell division of the sibling cell with Gaussian variation ranging from the mid phase of the corresponding sibling cell to that of the daughter cell (cell cycle index of 1.12±0.29 for groupA and 1.05±0.18 for groupB), regardless of differential growth conditions (Figure S5C). Note that transient upregulation of *hetR* expression in both h- and v-cells was hardly observed before the mid phase of the v-cell (data not shown), further supporting the mutual coupling of cell division cycle and developmental processes [16], [17].

These observations further indicate, at the single-filament level, that the impact of initial fluctuations in the magnitude of basal *hetR* expression and the photosynthetic activity represented by phycobilisome fluorescence on the determination of *de novo* (leading) proheterocyst positions is negligible. They also support the notion that the positions are selected at a later stage through dynamic interactions between cells [1], [18]. It is not surprising that dynamic cell–cell interactions are important in establishing the proheterocyst positions (developmental commitment [19]), because some competitive selection or lateral inhibition between cells is required [20], [21]. Meeks and Elhai [1] proposed a two-stage model of pattern formation in which four contiguous (synchronous) cells at similar favorable stages in their cell cycles start to differentiate after nitrogen step-down and then undergo competitive resolution to select a single heterocyst. In previous reports without time-lapse single-filament observations, contiguous cells expressing *hetR* (personal communication cited in Ref. 1) have been observed before the timing of expression confined to heterocysts, suggesting that single heterocysts are selected from such contiguous cells. We also observed two or more contiguous cells exhibiting bright *hetR::gfp* signals under liquid culture (Fig. S4), microchamber (Movies S1 and S2) and solid (Movies S3 and S4) conditions. It should be noted, as described above, that in most cases judged from cell lineage analysis, some of such contiguous cells with *hetR::gfp* upregulation are also possible to be the result of the division of the bright mother cells, and the fluorescence levels are often simultaneously reduced so that no single heterocyst develops from the contiguous cells. Interestingly, following the division of the cells displaying transient *hetR* upregulation, we often observed an enhanced P*hetR* signal in one of the neighboring cells leading to differentiation in our time-lapse analysis (e.g., the cell marked with a blue arrowhead in Figures 2C and 2D; see also Movies S1 to [Supplementary-material pone.0007371.s009] [Supplementary-material pone.0007371.s010] S4). Thus, in addition to the two-stage model, an additional competitive process is likely to be involved for proheterocyst positioning through cell-cell interactions.

Genetic approaches have revealed some inhibitors that are important in heterocyst patterning, such as the HetR-binding small peptide, PatS, which acts in *de novo* patterning [5], [10], [11], and HetN, which acts in the later stage to maintain the proper heterocyst intervals [12], [13]. The inactivation of both *patS* and *hetN* genes leads to the differentiation of nearly all the cells of a filament in the absence of compound nitrogen [13]. Moreover, small nitrogen compounds produced by committed heterocysts have also been suggested to act as diffusible inhibitors [1], [18], [22]. Importantly, the upregulation of the *patS* promoter activity in broader contiguous cells before the establishment of *de novo* heterocyst positions has been observed [11]. Cell lineage analysis with time-lapse single-filament observations of *patS* gene expression will be useful in validating whether a single heterocyst is selected from the contiguous cells, from neighboring cell(s), or from both. Moreover, time-lapse monitoring of *hetR* expression profiles in some strains with mutant inhibitor genes, leading to multicontiguous heterocysts [10], [12] at the single-filament level, should also provide insights into the dynamics of heterocyst development.

## Materials and Methods

### Bacterial strains and culture

*Anabaena* (*Nostoc*) sp. PCC 7120 and its derivative, SRhetR-1Gn, which harbors a transcriptional fusion of a promoterless *gfp* gene fused to the promoter of *hetR* (see below) were grown in 100 ml of BG-11 or BG-11_0_ (lacking sodium nitrate) medium in 200 ml flasks at 30°C under illumination with white fluorescent lamps at 45 µM photons m^–2^ s^–1^. The culture was bubbled with air (normal CO_2_). For synchronous heterocyst induction on plates, liquid cultures with an optical density at 730 nm (OD_730_) of about 0.2 were washed four times with BG11_0_, diluted to an OD_730_ of ∼0.01, and underlain beneath a fresh BG-11_0_ plate with or without fabricated micro-wells. For observation of heterocyst development in a liquid culture (Figure S4), SRhetR-1Gn was grown in BG-11 medium at 30°C under continuous illumination with a fluorescent lamp at 30 µM photons m^−2^ s^−1^. Liquid cultures were bubbled with air containing 1% (v/v) CO_2_. Filaments of SRhetR-1Gn grown in the presence of nitrate until they reached an OD_750_ of 0.4–0.5 were subjected to nitrogen deprivation. The transcriptional reporter *hetR*-*gfp* plasmid used for the SRhetR-1Gn strain was constructed as follows. A DNA fragment containing the *hetR* gene was amplified by polymerase chain reaction (PCR) using the primer pair hetR-uF (5′–TGCCAATGCAGAAGGTTAAA–3′) and hetR-RK (5′–TAGGTACCTCACTCTGGGTGCTTAATCTTC–3′) and cloned into the *Eco*RV site of pPCR-Script Amp SK(+) (Stratagene, La Jolla, CA, USA) to construct pPhetR. A *Bgl*II–*Hin*dIII fragment from pRL161 containing a neomycin-resistance cassette [23] was blunted and cloned into the blunt-ended *Hin*dIII site of the plasmid pKEN2-GFPmut2 [24]. The *gfp* gene and the neomycin-resistance cassette were excised together as an *Xba*I fragment from the resultant plasmid and inserted into the unique *Xba*I site located at 162 bases downstream of the 5′ end of *hetR* in pPhetR. A *hetR-gfp* transcriptional fusion gene was excised as an *Xho*I–*Sac*I fragment and was cloned between the *Xho*I and *Sac*I sites of pRL271 to construct pRhetRG. pRhetRG was transferred by conjugation into *Anabaena* PCC 7120 and a single recombinant, SRhetRG, was selected on a BG-11 plate containing neomycin and erythromycin.

### Microchamber arrays

To fabricate the microchamber array, a silicon mold was fabricated using MEMS technology. After dicing a 500 µm-thick silicon wafer (100, Waka Tech, Tokyo, Japan) into 20 mm×20 mm pieces, the substrate was spin-coated using a positive photoresist (TSMR-V90, Tokyo Ohka Kogyo, Japan) and patterned into an array of 200×200 µm microchambers by photolithography. The pattern is etched 8 µm in depth by deep reactive ion etching (Surface Technology Systems, Newport, Gwent, UK) (Figure S1A). Then, BG-11_0_ medium containing 1.5% agar solution was poured onto a silicon mold that was placed on a sterile plate to produce patterned solid medium with microwells on the bottom (Figure S1B). After the unnecessary agar was removed (indicated with dashed lines in Figure S1B), the patterned agar was placed onto an aliquot (∼50 µL) of prewashed *Anabaena* cell suspension placed on the bottom of the plate so that each single filament was stochastically enclosed in a microcage between the agar well and the surface of the plate (Figure S1B). To avoid desiccation, the patterned agar block was surrounded by fresh agar blocks on the plate (pale blue portions on both sides of the patterned block indicated in Figure S1B, bottom panel).

### Time-lapse monitoring system

BG-11_0_ plates were placed in a thermostat chamber (Microscope Incubation System, Tokai Hit, Shizuoka, Japan) kept at 30°C on an Olympus IX-71 inverted microscope (20× objective LUCPlanFLN lens, NA 0.45, Olympus, Tokyo, Japan) modified so that at least a 10-point analysis was possible with an automated X–Y stage controlled by a MAC 5000 automation controller system (Ludl Electronic Products Ltd., Hawthorne, NY, USA). Exchange of lamps, mirror-units, and sample-positions were programmed with Slidebook 4.1 (Intelligent Imaging Innovations, Denver, CO, USA) or Metamorph (Olympus) software. For bright field and fluorescence microscopy, we used a chilled charge-coupled device (CCD) camera (PIXIS1024, Princeton Instruments, Trenton, NJ, USA; or iXon^EM^+, Andor Technology PLC, Belfast, Northern Ireland) controlled using the software as above. Cells were grown under the microscope using white fluorescence lamps (FL30SW-B, 50 µEm^–2^ s^–1^; Hitachi Co., Tokyo, Japan) at 30 °C. Green fluorescent protein (GFP) and phycobilisome fluorescence during synchronous heterocyst development were monitored using filter sets U-MNIBA3 (Olympus) and U-MWIG3 (Olympus), respectively. The fluorescence excitation light intensity was attenuated with neutral density filters to avoid GFP fluorescence bleaching as described by Aldea *et al*. [15]. However, there was a tendency that the more repetitively irradiation of excitation lamps was given, especially to multipoint analysis which required, the more cell propagation and cell differentiation was likely to be delayed as discussed in the text. As a reference, we also monitored cells grown beneath solid agar plate without microchamber wells with lesser irradiation to observe P*hetR::gfp* signals without phycobilisome fluorescence (Movie S2C) and only morphological changes only (Movie S2D). Filaments grown under these conditions were often distorted to be fragmented or bleached, and the filaments shown in Movies S2C and S2D were exceptional samples that completed differentiation, while some cell size expansion and filament distortion were observed. For quantitative analysis, initially we used fluorescence beads (GE Healthcare, Little Chalfont, Buckinghamshire, UK) as external standards. However, they sometimes show decayed fluorescence profiles. Thus, for data shown in this manuscript, fluorescence intensity was normalized to the background signal for each image. For data shown in Figures 3C and 3D, fluorescence intensity was normalized globally such that the mean value per cell was 1.0.

## Supporting Information

Figure S1 (A) Schematic representation of the preparation of a silicon mold and a scanning electron microscopy (SEM) image used for preparing liquid microchambers from agar. (B) Schematic representation of agar wells made with a silicon mold as template and of the microscopic observation of *Anabaena* filaments enclosed in the microliquid spaces between the patterned agar and the culture plate. For more details, see the text. (C) Microchambers on the microscope. The dashed square at the top indicates the space of a microchamber. Some short *Anabaena* filaments are located in the first, third, and forth chambers. Bar, 100 µm.
(1.97 MB TIF)

Figure S2 (A, B) Cell lineages representing heterocyst differentiation in two individual *Anabaena* filaments grown in the microchambers. Magnification of the same cell lineage shown in Figure 2A (A) and that from a different filament (B). (C, D) Cell lineages from two individual filaments grown beneath flat solid medium. Note that in panel D, the two cells numbered 12 and 39 indicated at the right differentiated into heterocysts without cell division. Number 12 cell differentiated into one of the leading heterocysts (∼22 h after nitrogen deprivation).
(0.18 MB PDF)

Figure S3 Spatiotemporal dynamics of *hetR* expression profile monitored by the *gfp* reporter superimposed into the same cell lineages shown in Figures S2A (A) and S2C (B). Red bars at the right indicate cells that differentiated into heterocysts.
(0.50 MB PDF)

Figure S4 Morphological changes and P*hetR::gfp* fluorescence profiles from *Anabaena* cultures grown in liquid media after nitrogen step-down for 0, 3, 5, 8, 12 and 24 h.
(2.22 MB TIF)

Figure S5 (A, B) Intervals (numbers of vegetative cells in the filament) between heterocysts were not always regular at the initial stage of heterocyst differentiation, whereas they became more regular at the later stage so that heterocysts were found at about every 10 cells (filled circles). The cell lineage analysis also enabled us to plot intervals between mature heterocysts and a differentiating, plausible proheterocyst (white circle). The abscissa and ordinate indicate heterocyst intervals and time (h) after nitrogen step-down, respectively. Data for panels A and B were prepared from individual filaments whose cell lineages are shown in Figures S2A and S2C, respectively; ‘x’ indicates the distance between the leading heterocyst to both termini of the filaments. (C,) Profiles of cell propagation in the two filaments shown in Figures S2A (blue) and S2C (red). Arrows indicate timing of appearance of the leading heterocysts at the end of logarithmic growth (solid lines).
(0.13 MB TIF)

Figure S6 Transitional P*hetR::gfp* and phycobilisome fluorescence signals in cells from an individual Anabaena filament during the course of heterocyst formation at 42–72 h after nitrogen step-down. Cells were categorized into four groups, whose progenies (or themselves) differentiated into heterocyst(s): (1) at 62 h after nitrogen step-down (the leading heterocyst, open red circles); (2) at 63–65 h (during the transition state; filled red circles); (3) at 66–72 h (after establishment of regular patterns; filled orange circles), and (4) remaining vegetative cells at 72 h (filled green circles). It took ∼6 h between upregulation of the P*hetR::gfp* signal and reduction of phycobilisome fluorescence in each (pro)heterocyst. Importantly, upregulation of *hetR* gene expression was observed not only in heterocyst-forming cells but also transiently in vegetative cells.
(0.16 MB TIF)

Figure S7 Correlation of cell division and differentiation. (A) Total 13 transiently *hetR*-upregulating cells without leading to differentiation were compared with their sibling cells in time to divide from cell division of each mother cell (h). (B–D) Total 29 sets of sibling cells, one of which developed into heterocyst, were extracted from the lineages shown in Figure S2A (24 sets termed group A, microchambers) and S2C (5 sets termed group B, solid media). In each histogram, open and filled bars indicate cells in groups A and B, respectively. Timing (h) of cell division of the sibling v-cells (see text) after the corresponding mother cell division was scored (B). Timing (h) of cell differentiation of the h-cells (see text) after division of the mother cell was scored (C). Timing of cell differentiation in each h-cell was scored against the phase of normalized cell cycle of the sibling cell (cell cycle index; time 0 and 1 were defined as the timing of cell division in each mother cell and each v-cell, respectively) (D).
(0.18 MB TIF)

Movie S1 Time-lapse observations of morphological changes (bright-field, left), P*hetR::gfp* signals (middle), and phycobilisome fluorescence (right) in the filament grown under the microchamber condition shown in Figure S2A. Numbers indicate time (h) after nitrogen step-down. Bar, 45 µm
(9.34 MB MOV)

Movie S2 Time-lapse observations of morphological changes (bright-field, left), P*hetR::gfp* signals (middle), and phycobilisome fluorescence (right) in the filament grown under the microchamber condition shown in Figure S2B. Numbers indicate time (h) after nitrogen step-down. Bar, 45 µm
(7.59 MB MOV)

Movie S3 Time-lapse observations of morphological changes (bright-field, left), and P*hetR::gfp* signals (right) in the filament grown beneath a flat solid agar plate shown in Figure S2C. Numbers indicate time (h) after nitrogen step-down. Bar, 45 µm
(1.85 MB MOV)

Movie S4 Time-lapse observations of morphological changes (bright-field, left) in the filament grown beneath a flat solid agar plate shown in Figure S2D. Numbers indicate time (h) after nitrogen step-down. Bar, 50 µm
(0.40 MB MOV)
