# Supplementary figures and images for: Cyanobacterial Cell Lineage Analysis of the Spatiotemporal hetR Expression Profile during Heterocyst Pattern Formation in Anabaena sp. PCC 7120
Source: PLoS One. 2009 Oct 12;4(10):e7371. doi: 10.1371/journal.pone.0007371 (PMC2756587; doi:10.1371/journal.pone.0007371)

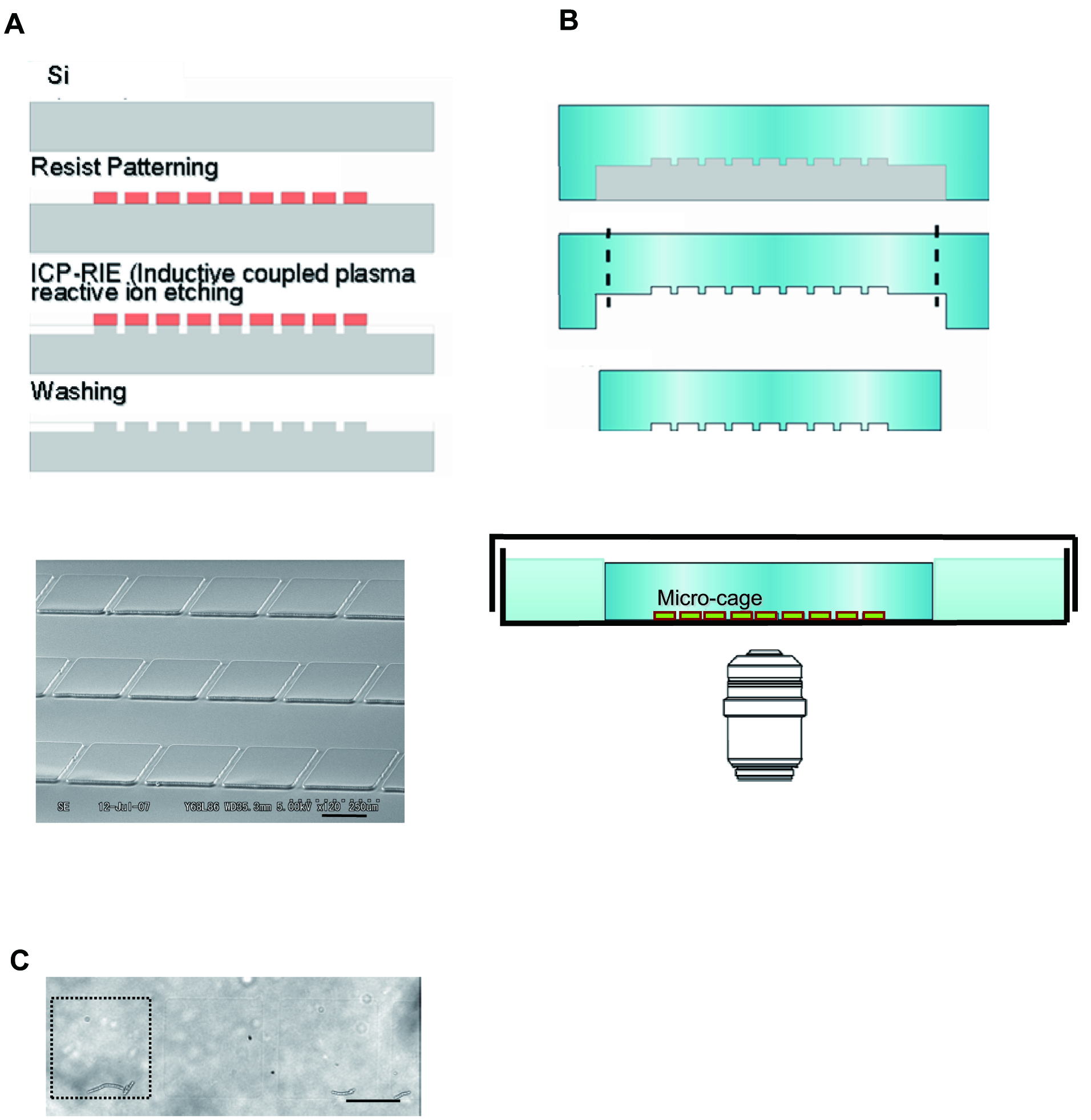

Supplement: Figure S1 — (A) Schematic representation of the preparation of a silicon mold and a scanning electron microscopy (SEM) image used for preparing liquid microchambers from agar. (B) Schematic representation of agar wells made with a silicon mold as template and of the microscopic observation of Anabaena filaments enclosed in the microliquid spaces between the patterned agar and the culture plate. For more details, see the text. (C) Microchambers on the microscope. The dashed square at the top indicates the space of a microchamber. Some short Anabaena filaments are located in the first, third, and forth chambers. Bar, 100 µm. (1.97 MB TIF) [file pone.0007371.s001.tif]

Fig. S2

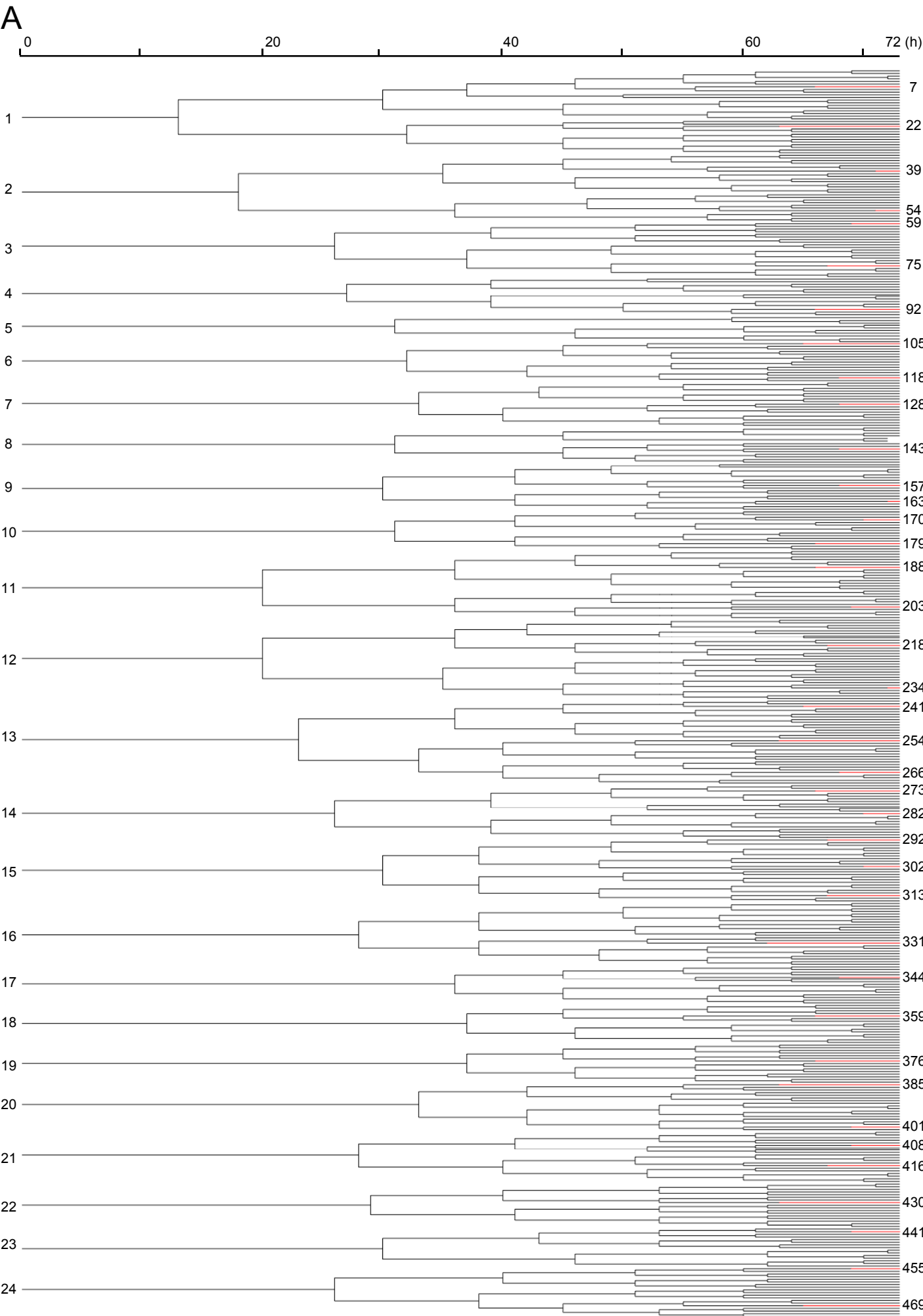

Fig. S2

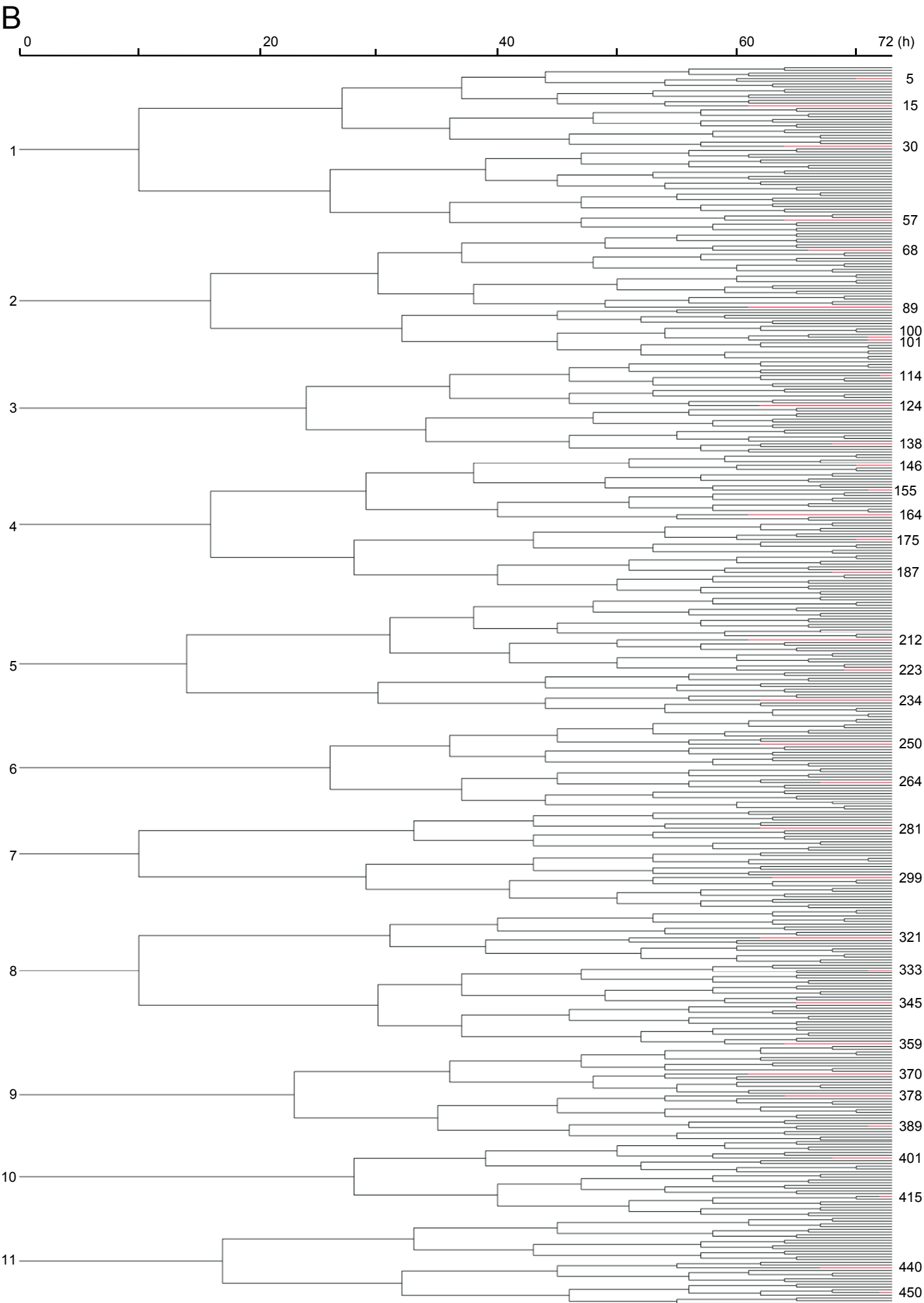

Fig. S2  
C

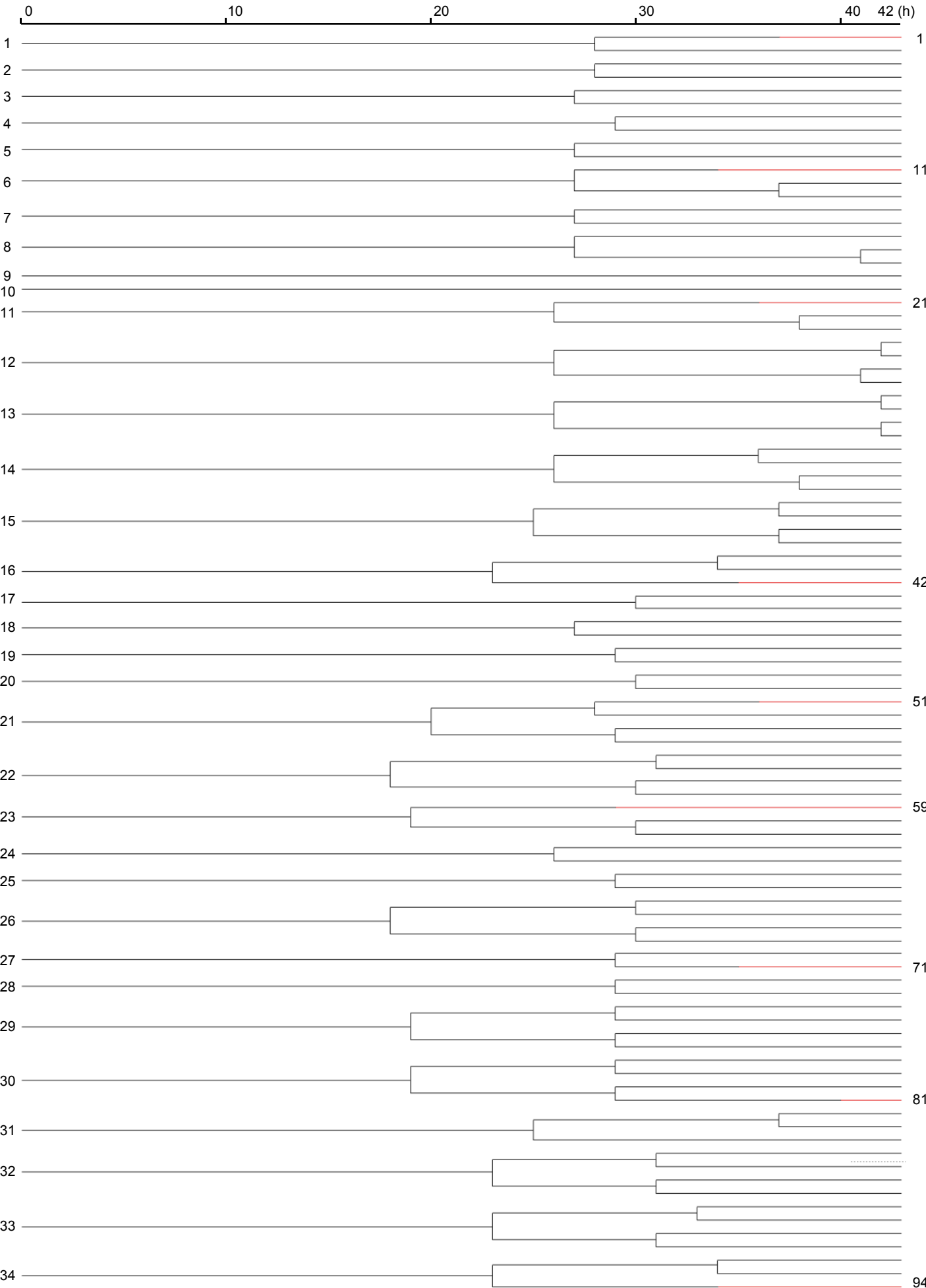

Fig. S2

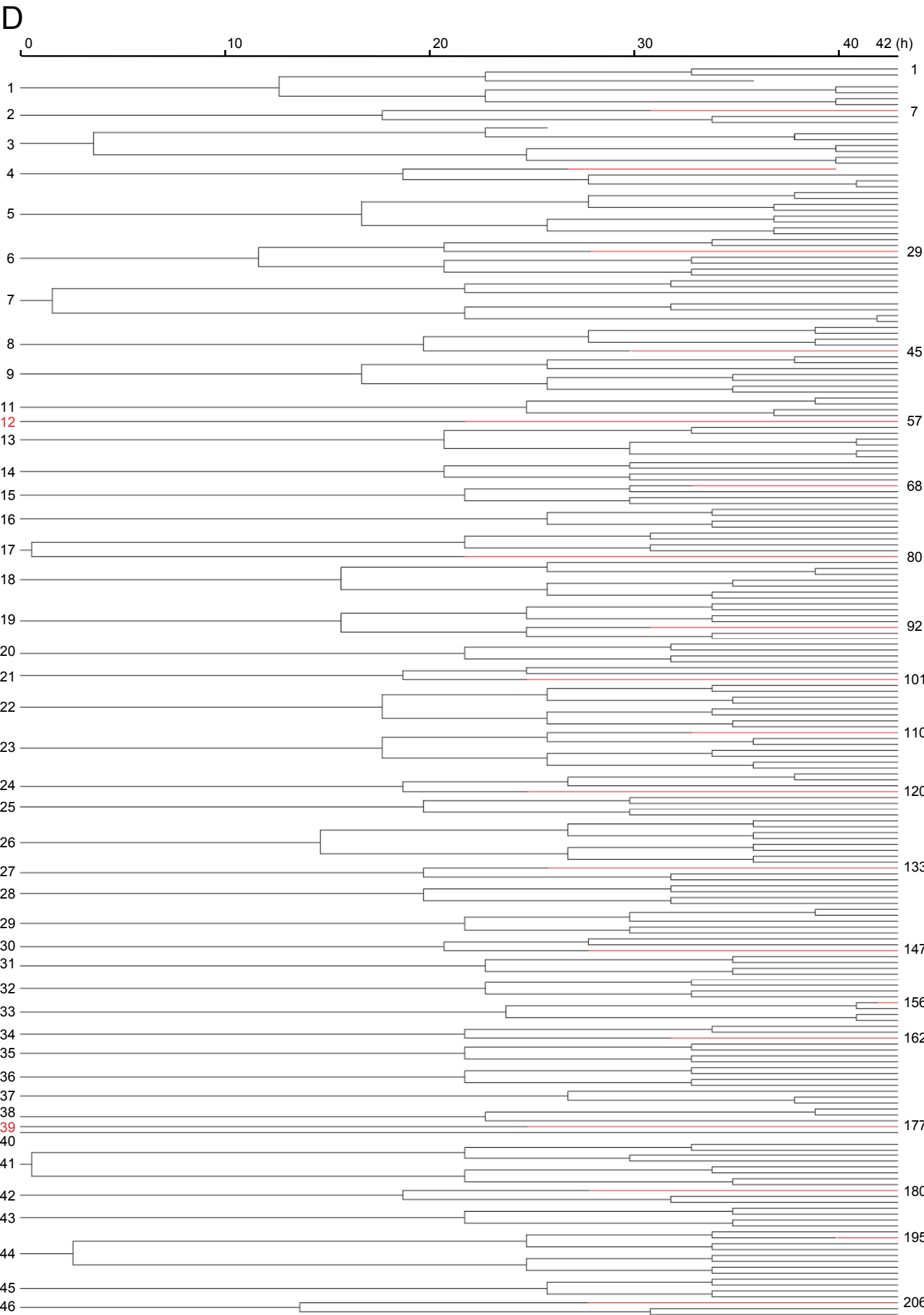

Supplement: Figure S2 — (A, B) Cell lineages representing heterocyst differentiation in two individual Anabaena filaments grown in the microchambers. Magnification of the same cell lineage shown in Figure 2A (A) and that from a different filament (B). (C, D) Cell lineages from two individual filaments grown beneath flat solid medium. Note that in panel D, the two cells numbered 12 and 39 indicated at the right differentiated into heterocysts without cell division. Number 12 cell differentiated into one of the leading heterocysts (∼22 h after nitrogen deprivation). (0.18 MB PDF) [file pone.0007371.s002.pdf]

Fig. S3

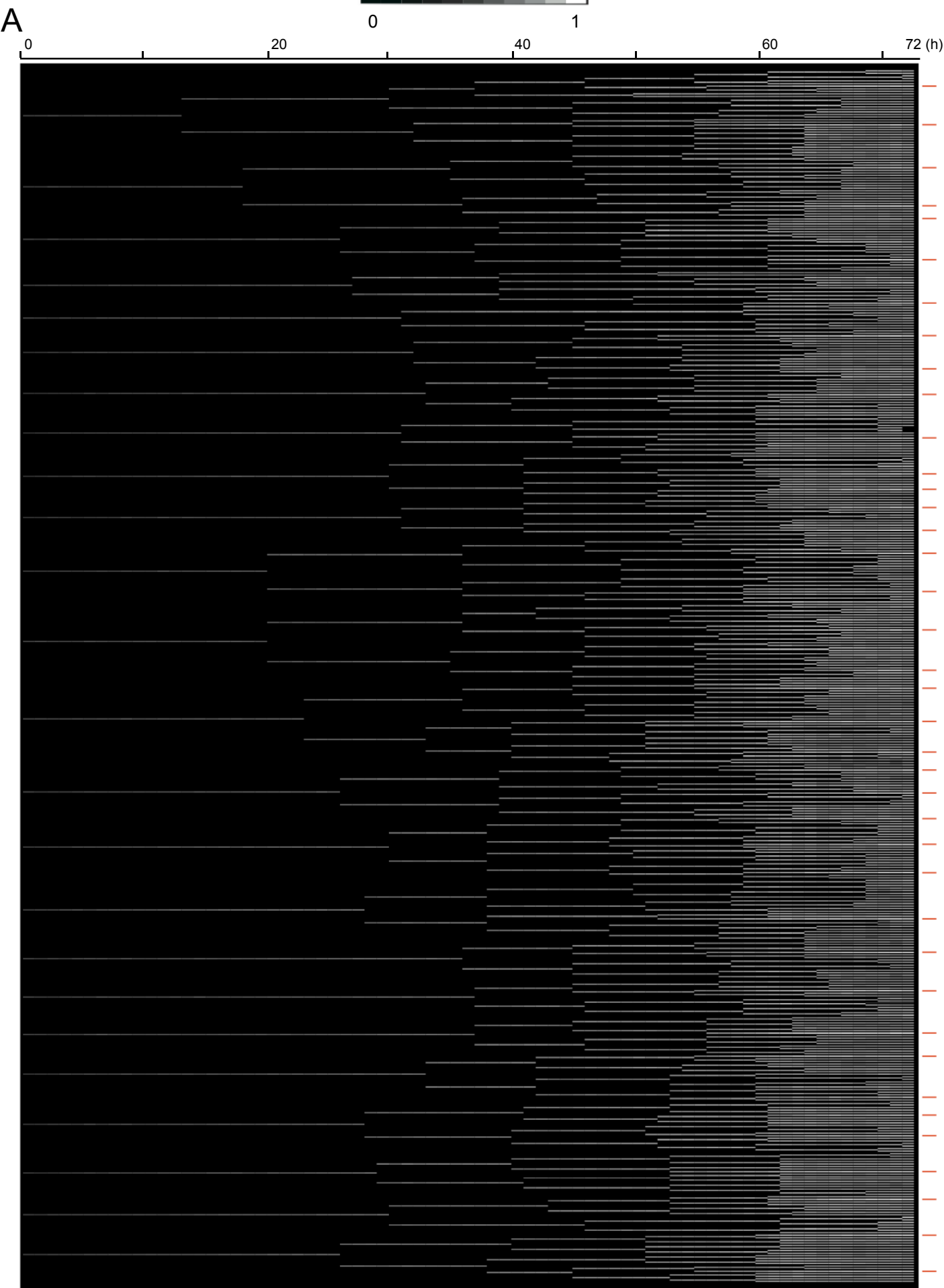

Fig. S3  
B

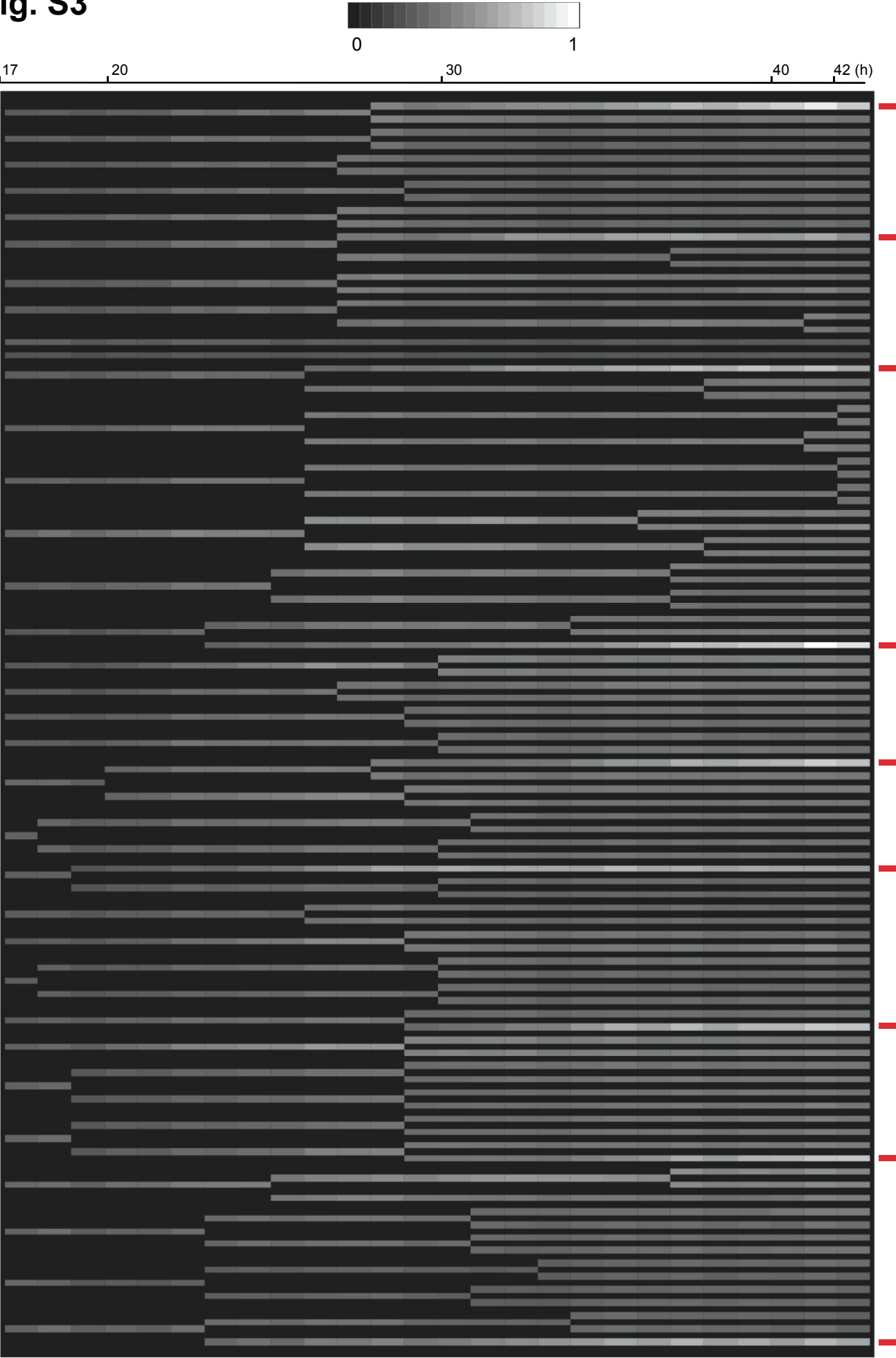

Supplement: Figure S3 — Spatiotemporal dynamics of hetR expression profile monitored by the gfp reporter superimposed into the same cell lineages shown in Figures S2A (A) and S2C (B). Red bars at the right indicate cells that differentiated into heterocysts. (0.50 MB PDF) [file pone.0007371.s003.pdf]

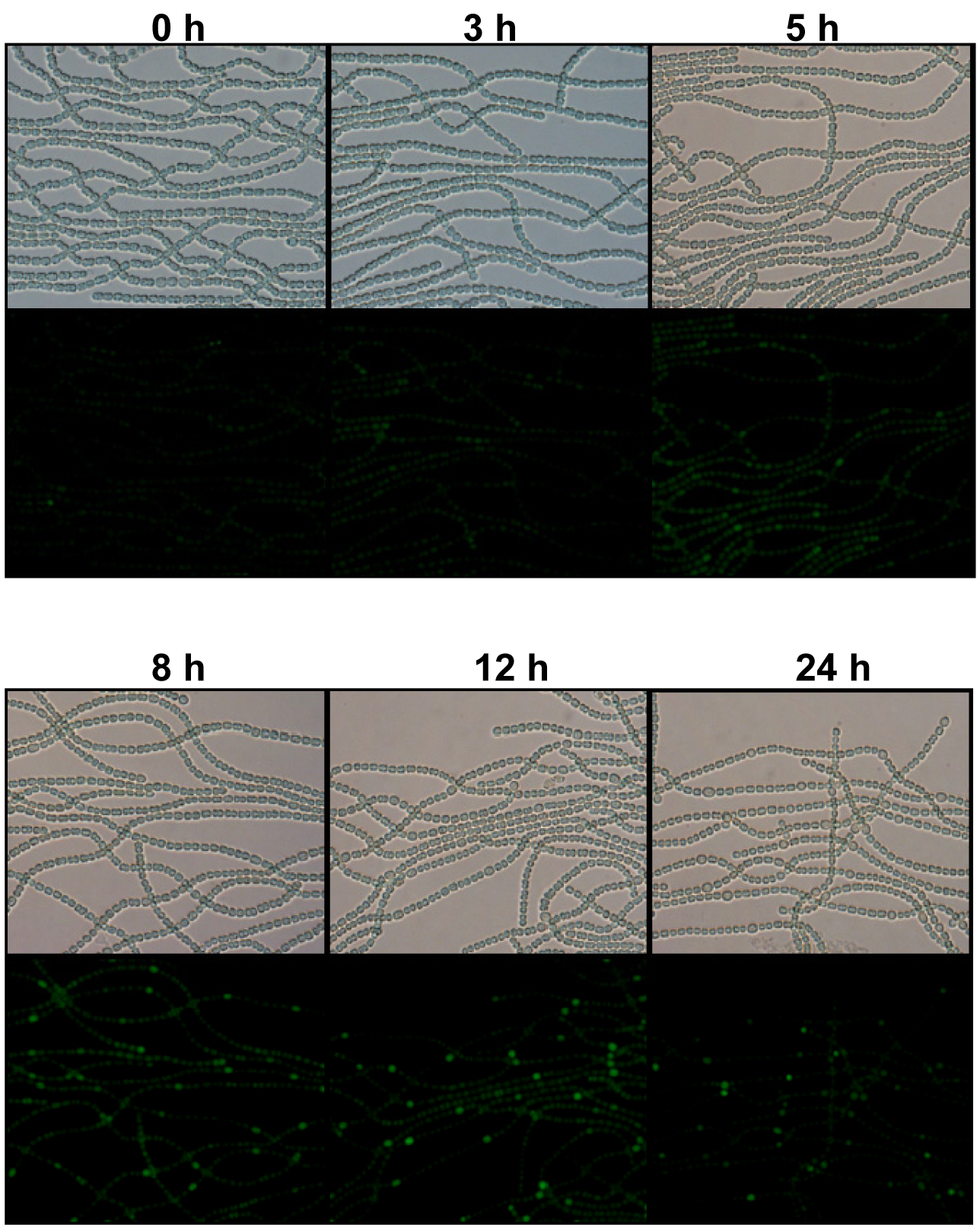

Supplement: Figure S4 — Morphological changes and PhetR::gfp fluorescence profiles from Anabaena cultures grown in liquid media after nitrogen step-down for 0, 3, 5, 8, 12 and 24 h. (2.22 MB TIF) [file pone.0007371.s004.tif]

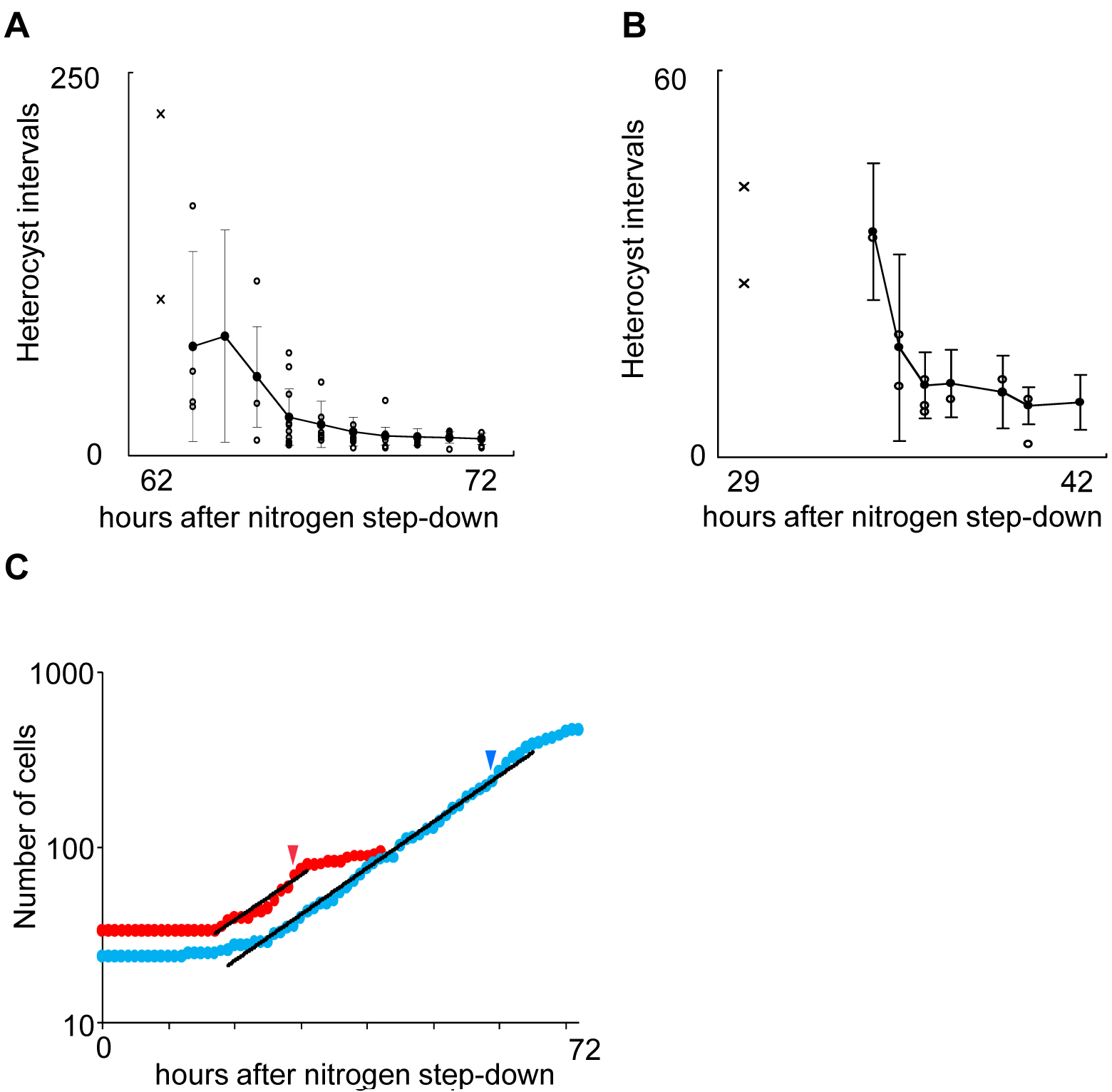

Supplement: Figure S5 — (A, B) Intervals (numbers of vegetative cells in the filament) between heterocysts were not always regular at the initial stage of heterocyst differentiation, whereas they became more regular at the later stage so that heterocysts were found at about every 10 cells (filled circles). The cell lineage analysis also enabled us to plot intervals between mature heterocysts and a differentiating, plausible proheterocyst (white circle). The abscissa and ordinate indicate heterocyst intervals and time (h) after nitrogen step-down, respectively. Data for panels A and B were prepared from individual filaments whose cell lineages are shown in Figures S2A and S2C, respectively; ‘x’ indicates the distance between the leading heterocyst to both termini of the filaments. (C,) Profiles of cell propagation in the two filaments shown in Figures S2A (blue) and S2C (red). Arrows indicate timing of appearance of the leading heterocysts at the end of logarithmic growth (solid lines). (0.13 MB TIF) [file pone.0007371.s005.tif]

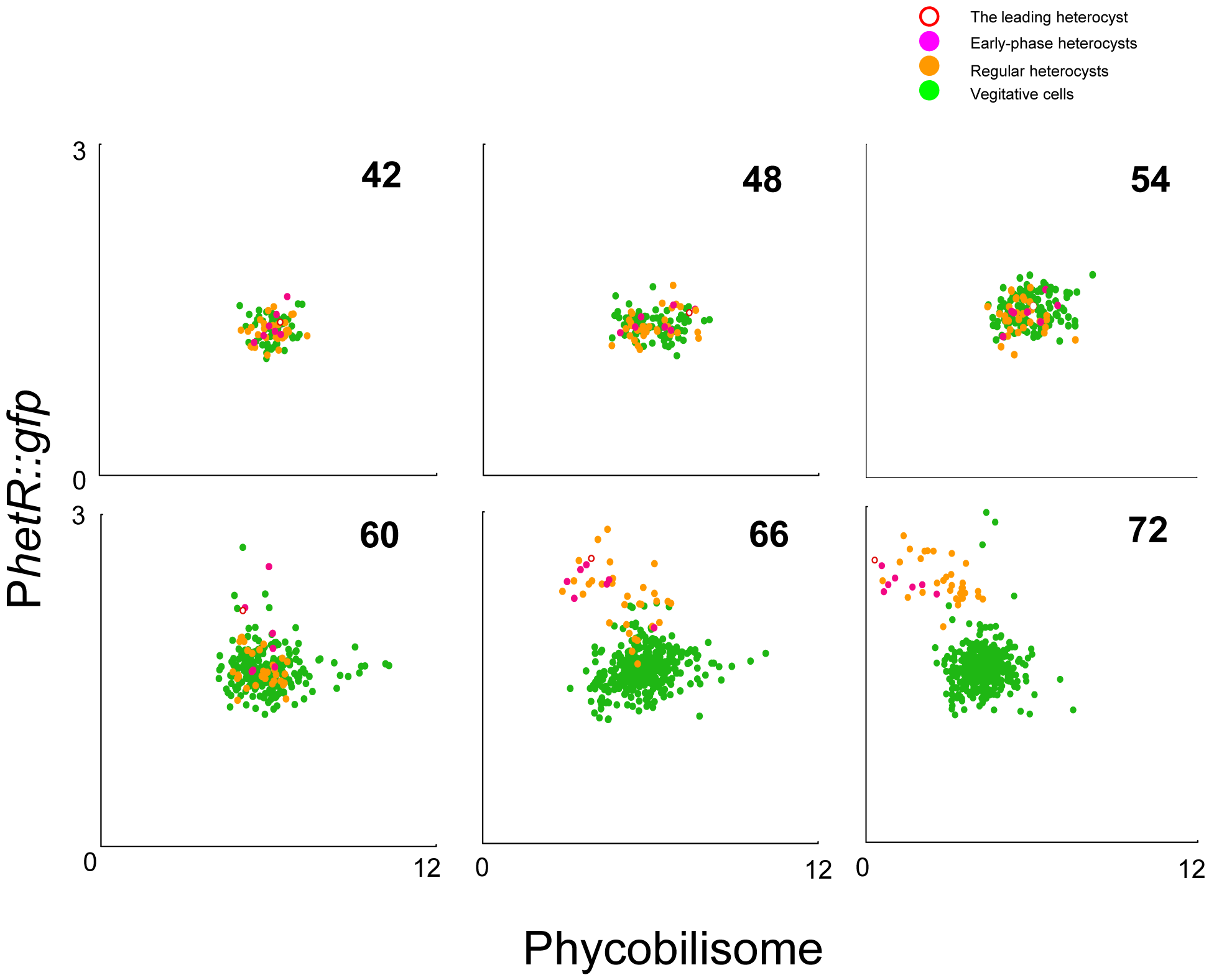

Supplement: Figure S6 — Transitional PhetR::gfp and phycobilisome fluorescence signals in cells from an individual Anabaena filament during the course of heterocyst formation at 42–72 h after nitrogen step-down. Cells were categorized into four groups, whose progenies (or themselves) differentiated into heterocyst(s): (1) at 62 h after nitrogen step-down (the leading heterocyst, open red circles); (2) at 63–65 h (during the transition state; filled red circles); (3) at 66–72 h (after establishment of regular patterns; filled orange circles), and (4) remaining vegetative cells at 72 h (filled green circles). It took ∼6 h between upregulation of the PhetR::gfp signal and reduction of phycobilisome fluorescence in each (pro)heterocyst. Importantly, upregulation of hetR gene expression was observed not only in heterocyst-forming cells but also transiently in vegetative cells. (0.16 MB TIF) [file pone.0007371.s006.tif]

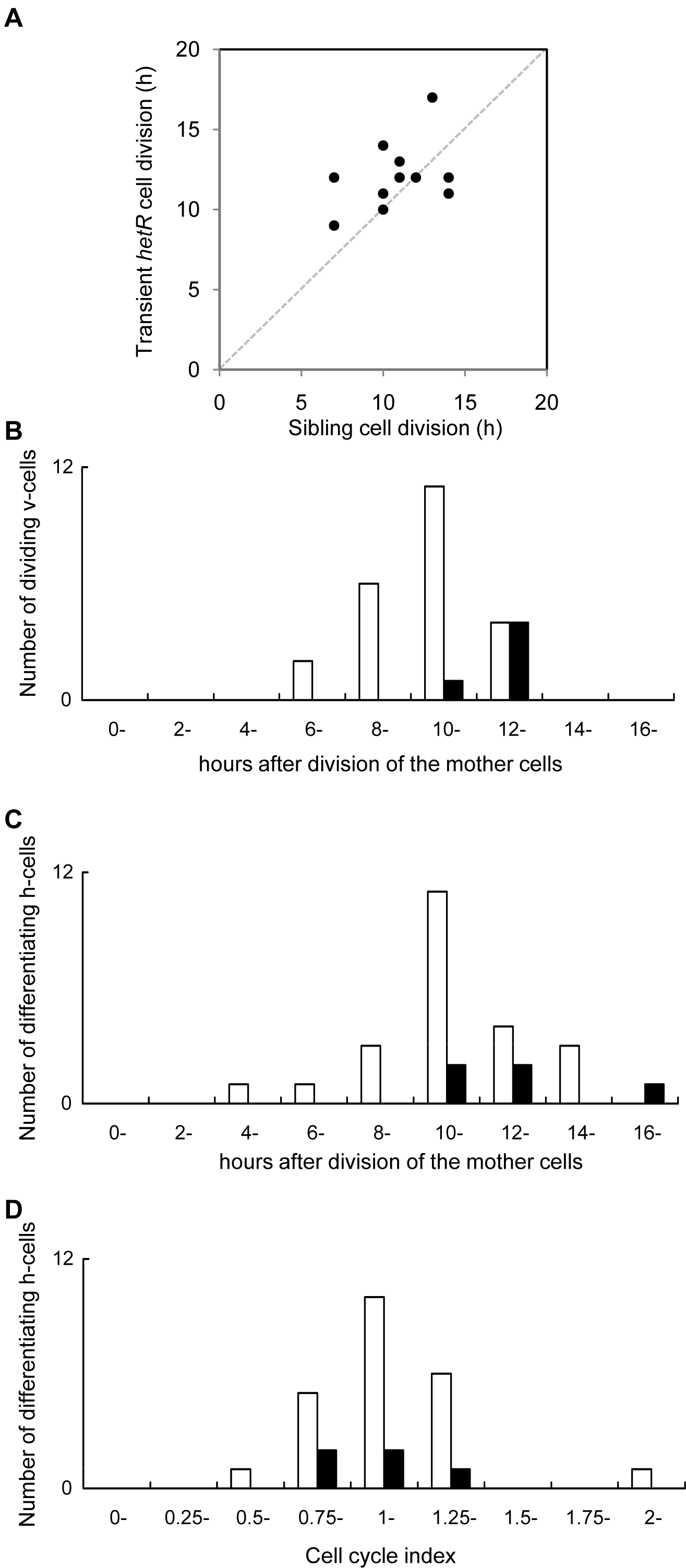

Supplement: Figure S7 — Correlation of cell division and differentiation. (A) Total 13 transiently hetR-upregulating cells without leading to differentiation were compared with their sibling cells in time to divide from cell division of each mother cell (h). (B–D) Total 29 sets of sibling cells, one of which developed into heterocyst, were extracted from the lineages shown in Figure S2A (24 sets termed group A, microchambers) and S2C (5 sets termed group B, solid media). In each histogram, open and filled bars indicate cells in groups A and B, respectively. Timing (h) of cell division of the sibling v-cells (see text) after the corresponding mother cell division was scored (B). Timing (h) of cell differentiation of the h-cells (see text) after division of the mother cell was scored (C). Timing of cell differentiation in each h-cell was scored against the phase of normalized cell cycle of the sibling cell (cell cycle index; time 0 and 1 were defined as the timing of cell division in each mother cell and each v-cell, respectively) (D). (0.18 MB TIF) [file pone.0007371.s007.tif]
